# Mesoporous Silica-Based Materials for Electronics-Oriented Applications

**DOI:** 10.3390/molecules24132395

**Published:** 2019-06-28

**Authors:** Łukasz Laskowski, Magdalena Laskowska, Neus Vila, Mateusz Schabikowski, Alain Walcarius

**Affiliations:** 1 Institute of Nuclear Physics Polish Academy of Sciences, PL-31342 Krakow, Poland; 2 Laboratoire de Chimie Physique et Microbiologie pour les Matériaux et l’Environnement (LCPME), UMR 7564 CNRS—Université de Lorraine, 405 rue de Vandoeuvre, 54600 Villers-les-Nancy, France

**Keywords:** mesoporous silica materials, electrodes, supercapacitors, low-*k* dielectrics, sensors, molecular electronics, functionalized silica, electron transfer

## Abstract

Electronics, and nanoelectronics in particular, represent one of the most promising branches of technology. The search for novel and more efficient materials seems to be natural here. Thus far, silicon-based devices have been monopolizing this domain. Indeed, it is justified since it allows for significant miniaturization of electronic elements by their densification in integrated circuits. Nevertheless, silicon has some restrictions. Since this material is applied in the bulk form, the miniaturization limit seems to be already reached. Moreover, smaller silicon-based elements (mainly processors) need much more energy and generate significantly more heat than their larger counterparts. In our opinion, the future belongs to nanostructured materials where a proper structure is obtained by means of bottom-up nanotechnology. A great example of a material utilizing nanostructuring is mesoporous silica, which, due to its outstanding properties, can find numerous applications in electronic devices. This focused review is devoted to the application of porous silica-based materials in electronics. We guide the reader through the development and most crucial findings of porous silica from its first synthesis in 1992 to the present. The article describes constant struggle of researchers to find better solutions to supercapacitors, lower the *k* value or redox-active hybrids while maintaining robust mechanical properties. Finally, the last section refers to ultra-modern applications of silica such as molecular artificial neural networks or super-dense magnetic memory storage.

## 1. Introduction

Electronics can be considered as the field of the solid-state physics dealing with electron transport phenomenon in vacuum, gases and solids [1]. Considering these criteria, we can divide it into vacuum electronics (this domain includes emission, the flow and control of electrons both in vacuum and in gases) and semiconductor electronics [2,3]. This strongly applicative science is continuously present in our life since 1905 when Lee De Forest invented the triode [4]. At that time, electronics were based mainly on electronic tubes and their main focus was radio broadcasting.

The years of World War II brought rapid development of novel electronic devices; indeed, 1940 is claimed to be the year of the invention of the resonant-cavity magnetron by Randall and Boot from Birmingham University [5]. Even though the history of magnetron was different from the way it is presented by the winners of the World War II [6], this invention was unquestionably extremely important since it allowed for the construction of radars [7]. In 1943, Rudolf Kompfner introduced the traveling-wave tube (which was a great improvement over Haeff’s similar device from 1931) [8,9,10]. It caused dynamic development of the microwave technique, which was mainly focused on radar technology. It was the time of the first attempts to miniaturize and improve the reliability of electronic devices.

After the World War II, electronics were still developing as a result of the formation of new branches of science and technology, mainly information technology. The era of semiconductors was opened by Shockley, Bardeen and Brattain with the development of a transistor (for which they were jointly awarded the Nobel Prize in Physics in 1956) [11]. The transistor was a slice of germanium and three carefully placed wires in contact with it [12]. The device was not only a valve but also an amplifier. Due to its advantages, such as a small size, durability, reliability, low supply voltage and the lack of a filament circuit, the transistor rapidly replaced the electronic tube. It quickly became a commonly used electronic device based on semiconductors. Nevertheless, the germanium transistors were unreliable and engineers started to search for new materials to construct those devices [13]. In their search, they discovered silicon which proved to be a better material for transistors. Thus, the silicon era had begun.

The material allowed for significant miniaturization of electronic elements by their compression in the integrated circuits [14]. From the 1960s, electronic devices became smaller and smaller. The rate of this miniaturization can be described by Moore’s law [15], which claims that the number of transistors in a dense integrated circuit doubles approximately every two years. Actually, presently, we should say that processing power doubles approximately every two years [16].

Artificial intelligence plays an increasingly important role in our lives by continually improving the quality of life [17,18]. This domain of computer science demands enormous computational power to allow real-time operation of neural-based systems. Presently, commercially available processing units reach the dimensions as small as less than 0.04 m^3^ [19]. However, it is still insufficient for real-time operation of those systems. A question arises: How small can electronic elements be? Can we simply trust in Moore’s law and wait? The answer is “no”. It seems that we are close to reaching the limit of silicon technology [20,21]. Decreasing the size of processing units causes an immense increase of their energy needs. Smaller silicon processors need much more energy than their larger counterparts and also generate significantly more heat [22]. Based on the history of technology, we can be sure that, by approaching this limit, we will provoke a technological breakthrough. Silicon will be substituted by a different material opening a wide perspective for further miniaturization. In our opinion, such a new material can be composed of nanostructures based on silica, allowing for building devices atom by atom by means of nanotechnology.

**Figure 1:**
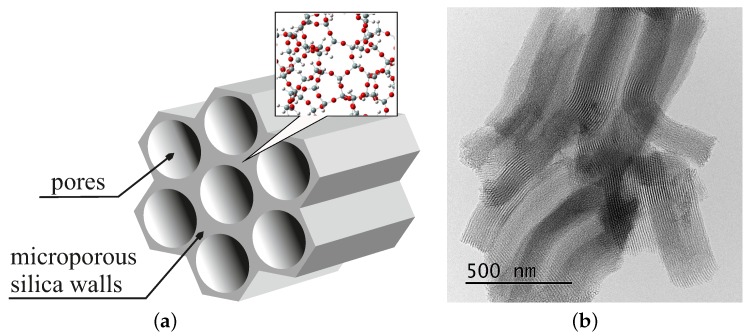
The structure of SBA-15 type mesoporous silica: a scheme (**a**); and a TEM micrograph (**b**).

Porous silica is a promising candidate for such applications. Since the first synthesis of MCM-41 mesoporous silica in 1992 (Mobil Composition of Matter No. 41) by Mobil Oil Corporation, this kind of material has aroused a lot of interest [23]. These silica-based systems have a specific structure: they posses a 2D hexagonal arrangement of cylindrical pores and their walls are made of amorphous silica gel [24]. The material exhibits a colossal specific surface area, typically approximately 1000 m^2^/g or even more [25]. Despite several advantages, such as a homogeneous pore distribution, an enormous specific surface area and the large volume of pores, their mechanical and chemical stability was usually evaluated as quite poor, especially in aqueous and alkaline media. However, many years of struggling to improve them were fruitful. In 1998, a next revolutionary material was developed: SBA-15 mesoporous silica (Santa Barbara Amorphous) [26,27]. This material has amorphous walls much thicker than the MCM-41 [28], resulting in improved stability even under humid conditions. It contains hexagonally-arranged cylindrical pores within amorphous microporous silica. SBA-15 has pores with a larger diameter than the MCM-41. It is also significantly more stable. This mesoporous material is fabricated with the use of Pluronic P123, a triblock copolymer, as a template. During the synthesis in an acidic environment, silica molecules enclose the non-ionic surfactant P123. After the removal of the surfactant, two types of pores form: (1) homogenous mesopores with the diameter from 4 to 40 nm (depending on synthesis route), which are from 200 nm up to a few μm long and are arranged hexagonally; and (2) larger micropores that connect mesopores with each other [29,30,31]. The structure of this material is depicted in Figure 1.

The material is inexpensive to synthesize and has numerous other advantages. It is characterized by highly uniform porosity, good mechanical stiffness, thermal stability, a high volume of pores and a large specific surface area, typically in the range of 400–900 m^2^/g [32]. The structural parameters of SBA-15, such as the diameter of pores, microporosity and the thickness of walls, can be modified by tuning parameters of the synthesis [33]. Since its discovery, it has remained one of the most popular porous matrices.

Nevertheless, mesoporous silica in its pure form drew the attention of scientists for only a short time. Its real potential lays in the possibility of its functionalization: both MCM and SBA architectures are excellent candidates for the fabrication of host–guest systems [34,35,36]. In particular, several anisotropic forms of porous silica, such as sheets [37,38,39] or thin films [40,41,42], are interesting from the perspective of their application. From the literature on the subject of the real practical applications of porous silica-based systems, the articles concerning electronics constitute the minority. This situation is thought-provoking since such material gives numerous possibilities for the creation of very precise nanostructures for the control of charge transport. In our opinion, porous silica has a massive applicative potential in this field. It was the reason we decided to review this domain and present a few perspectives for the application of silica in advanced electronic systems.

## 2. Supercapacitors

One of the most prominent electronic devices utilizing the porous silica-based materials are supercapacitors [43]. These devices can be described as electronic elements with parameters merging the features of capacitors and accumulators. They can accumulate significant amount of energy (their accumulated energy density is an order of magnitude smaller than accumulators, which is still even two orders of magnitude larger than conventional condensers) and, similar to capacitors (unlike rechargeable batteries), they can be charged and recharged swiftly, allowing to obtain large energies in a short time [44,45].

The supercapacitors consist of a few main components: current collectors, active electrodes, an electrolyte and a separator. Unlike conventional capacitors, they do not contain a dielectric layer. This role is played by the interface between an active electrode and an electrolyte. A porous membrane, of the order of a few Å, usually functions as a separator. It separates electrodes while still maintaining the transfer of free ions. Supercapacitors can be divided into two main types, depending on the mechanisms of charge storing.

**Figure 2:**
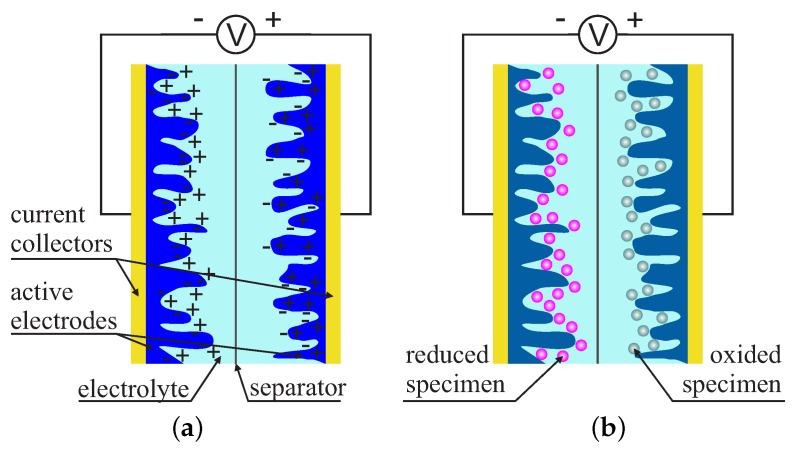
A schematic illustration of supercapacitors: an electrical double-layer capacitor (**a**); and a pseudocapacitor (**b**).

**Electrical double-layer capacitors (EDLC)** utilize purely electrostatic processes for the accumulation of charge on the interface between an electrolyte and an electrode. **Electrochemical pseudocapacitors** feature the Faradaic electron charge transfer: fast and reversible redox reactions at the surface of active materials [46,47]. The differences between mentioned types of supercapacitors are shown in Figure 2.

Looking at Figure 2, one can easily conclude that the surface of active electrodes is crucial for the capacity of supercapacitors [48]. Indeed, the specific surface is extremely important for the parameters of these devices. It is also the reason scientists search for new materials for electrodes.

Nanomaterials with highly ordered nanostructure seem to be promising candidates for such applications [49,50], in particular carbon-based nanomaterials such as graphene, mesoporous carbon or carbon nanotubes [51,52,53,54,55,56] due to their immense specific surface area and electric conductivity. Nevertheless, their main limitation is their cost, which prevents commercial use. Similar structural properties can be readily tailored on mesoporous silica, which is much cheaper and easier to fabricate. However, silica deals with a significant problem: it is non-conductive.

**Figure 3:**
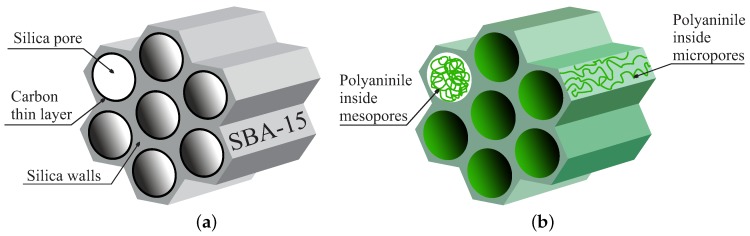
A schematic representation of the porous silica-based composite materials for the application of supercapacitors: (**a**) a carbon-lined SBA-15 mesoporous silica; and (**b**) SBA-15 containing polyaniline chains inside its structure.

To assure the conductivity of porous silica, their pores can be lined by a conductive material such as carbon. This approach was applied by Zhi et al. [57,58,59,60]. The basic material proposed by the authors is presented in Figure 3a.

The fabrication of these silica-supported carbon nanomembranes was possible by using Bergman cyclization of compounds containing enediyne immobilized inside SBA-15 nanochannels and by pyrolyzing the obtained compound. Since carbon lined SBA-15 silica has shown promising structural parameters, such as a homogenous arrangement of pores and a high specific surface area [57,58], it was used as a base material for electrodes for electrical double layer capacitors.

Both SBA-15 (also with some pore-expanded architectures [61,62]) and MCM-41 silica were lined by carbon and investigated regarding their electrochemical properties [59]. The authors obtained composite materials with pores varying from 3 to 8 nm and the specific surface area in the range from 190 to 450 m^2^/g. It is significantly less than for pure porous silica but such composite specimens are conductive. Moreover, all materials exhibited perfect capacitive behavior [63]: the obtained cyclic voltammetry curves were quasi-rectangular in shape along the current–potential axis. All of the materials showed excellent EDLC galvanostatic charge–discharge curves and short relaxation time constants. The specific capacitance was also considerable but varied significantly depending on the architecture of the materials. Interestingly, the specific capacitance dependence on the diameter of pores and the specific surface area was not monotonic. The maximum capacitance (305 F/g) was found for porous material with the pore size of 5.24 nm. It was significantly higher than that of other silica with either smaller or larger pores. Nevertheless, for all of the porous materials, the specific capacitance was substantial and each one of them is a promising candidate as a component of supercapacitors.

This great performance can still be enhanced by the inclusion of metal oxide nanorods inside the porous material with the subsequent removal of the silica matrix, as shown in [60]. Here, SBA-15 silica acts as a template to obtain 2D arranged nanorods inside the carbon layer. The nanorods of manganese oxide, tin oxide and nickel oxide inside SBA-15 porous silica lined with carbon were fabricated by means of impregnation by solution of the metal salts and subsequent calcination. Next, the silica matrix was removed by dissolving it in a basic solution. The obtained material was proposed to be applied in pseudocapacitors since transition metal oxides are recognized as ideal materials for Faradaic supercapacitor electrodes [64,65,66]. Indeed, the described materials showed great electrochemical features. They exhibited high specific capacitances, from 745 to 964 F/g with a good cycling stability—less than 10% capacitance decay over 10,000 circles. The energy densities varied between 21.6 and 33.5 Wh/kg. The authors ascribed such performances to two key features: the high electrical conductivity and an intimate contact between the carbon membrane and well-ordered metal oxide nanorods.

A different approach of making porous silica conductive by merging it with a conductive polymer, such as polyaniline (PANI), was presented in [67]. The proposed material is interesting since polyaniline resides both in silica pores and inside microchannels in the structure of silica walls. This configuration is shown in Figure 3b.

The structure was achieved by a synthesis which is actually a simplified and optimized route presented before by Silva and Asefa [68]. In the first step, SBA-15 silica platelets were impregnated by ammonium persulfate, then washed and dried. This step resulted in presence of ammonium persulfate inside both silica channels and wall structures: in micropores. In the next step, the polymerization of aniline was performed inside silica mesopores and micropores by impregnation with the aniline solution. It resulted in the presence of polyaniline inside both meso- and micropores. By closely examining the results of transmission electron microscopy and nitrogen sorption analysis, one comes to the conclusion that polyaniline chains resides inside mesopores mainly in close vicinity of the walls. In the TEM images, it is clearly seen that pores are patent. Similarly, nitrogen sorption analysis showed that pores are present in the material, however narrower than those in pure SBA-15 silica.

The electrochemical measurements showed that the material possesses great applicative potential in the field of supercapacitors. Applying SBA-15-polyaniline composite as an electrode allows for obtaining excellent electrochemical performance: the energy density was 1.6 F/g and power density was evaluated to be 173 Wh/kg at the discharge rate of 0.5 A/g (material was investigated in KI electrolyte solution within the potential range from −0.3 to 0.324 V) [68].

An example of merging both aforementioned materials is described in [69]. The authors presented a composite of MCM-41 porous silica in the form of nanospheres, graphene oxide and nanowires of PANI. However, the material had no ordered nanostructure but rather was just a mixture of the constituents. Interestingly, such an unordered structure enhanced the electrochemical properties of the pure PANI electrode: the specific capacitance reached 412 F/g at the current density of 1 A/g in comparison to 384 F/g for the pure PANI. Moreover, such an electrode has shown better stability than pure PANI. Nevertheless, in our opinion, the improvement over the PANI electrode was not significant and, thus, not worth such a complicated synthesis.

The porous silica can be treated just as a template for the fabrication of conductive materials in the form of rods or 2D ordered porous structures. Such a method was presented by Wang et al. [70]. The authors impregnated SBA-15 silica framework with the solution of Ni(NO_3_)·6H_2_O and, after drying and pyrolysis, the porous silica template was removed by etching in a solution of 2 M NaOH. As a result, 2D ordered NiO nanowires were obtained. Because the ordered silica was used as a template, the material has a porous structure with a shape that could considered as the negative shape of SBA-15 silica. The used technology increased the specific surface area and consequently enhanced the electrochemical parameters of the electrode. For the obtained material, the authors observed a significant increase of the specific capacitance in comparison to NiO prepared via the ordinary method. The specific capacitance was approximately 128 F/g, which is an increase of about 400%. This result is not spectacular, but definitely worth noting.

A similar approach was applied to obtain the manganese dioxide 2D ordered nanowires [71]. Here, Ghimbeu et al. presented a more complicated synthesis route. The KIT-6 mesoporous silica was used in the first step of fabrication of the lithium-manganese oxide nanowires, which were transformed, after silica etching, into manganese dioxide by hydrolysis in an aqueous solution of sulfuric acid. The authors obtained specific capacitance of up to 283 F/g, but very poor stability.

A somewhat reversed method was presented for conductive mesoporous carbon [72]. In this case, the synthesis of porous carbon was performed using SBA-15 silica and sucrose as the source of carbon. Dry SBA-15 was impregnated with an aqueous solution of sucrose containing sulfuric acid. The carbonization was performed by thermal treatment after drying. The porous silica template was removed by dissolution in a NaOH solution. This basic material can be further oxidized by utilizing a nitric acid to obtain materials for electrodes for supercapacitors [73]. The electrochemical measurements showed promising electrochemical parameters of the samples. The authors observed the specific capacitance in the range of 99–159 F/g (depending on the architecture, which in turn depends on the conditions of thermal treatment), while the energy density reached 5.7 Wh/kg.

Similar procedure was presented by Singh et al. [74], but in this case, the teflon-assisted ultrafast removal of silica was applied instead of sodium hydroxide solution. The obtained electrochemical parameters were better than as in the previous article—the specific capacitance was evaluated to be 292 F/g. Those results were not spectacular but significantly better than the characteristics of regular porous carbon.

The performance of materials obtained in the aforementioned articles can be improved. It can be realized by the functionalization of porous carbon, fabricated with the use of a SBA-15 template, by some metal oxides exhibiting Faradaic oxidations. Such an approach was applied by Zhi et al. in [75]. Co_3_O_4_ was immobilized inside the channels of the porous carbon. The authors claimed that the presented material is a composite of SBA-15 and carbon with Co_3_O_4_ nanoparticles attached to the inner walls of the channels. The SBA-15 silica was removed by soaking in an aqueous NaOH solution and, thus, it should be considered as mesoporous carbon functionalized with cobalt oxide nanoparticles. The obtained materials exhibited maximum specific capacitance of 1086 F/g in 6 M KOH solution and great electrochemical stability: the specific capacitance remained at 90% of the initial value after 10,000 consecutive cycles.

An analogous yet simplified material to this presented above was described by Huang et al. [76]. The authors applied mesoporous silica nanoparticles [77] as a template for hydrous ruthenium oxide (RuO_2_·H_2_O) thin layer. An evaporation-induced coating was applied and the procedure was followed with microwave-assisted hydrothermal transformation to obtain a hybrid material: porous silica lined with RuO_2_·H_2_O layer. The optimized material, regarding the content of RuO_2_·H_2_O, was characterized by excellent parameters for high-performance supercapacitors: the obtained specific capacitance was over 1000 F/g. These results are very important since the presented material integrates costly rare-earth elements with relatively inexpensive compounds for the economical fabrication of high-performance supercapacitors.

Judging from the amount of literature on the subject, one can conclude that porous silica-based electrodes for supercapacitors are in constant development.

## 3. Low-*k* Dielectrics for Electronic Devices

The dielectric constant κ, frequently denoted as a *k*, describes the response of the material to an external electric field. In other words, it is a measure of how an electric field affects, and is affected by, a dielectric material. It can be expressed in the form of Equation (1),

(1) $$\kappa =\frac{\varepsilon }{\varepsilon_{0}}$$

where ε is the complex permittivity of the material and $\varepsilon_{0}$ is the vacuum permittivity [78,79]. Both κ and ε are frequency-dependent parameters. For the frequency of 0 Hz, conductivity is the only contribution to dielectric constant.

The low-*k* dielectric materials (materials with *k* < 3) are crucial for the integrated circuit (IC) technology [80,81]. In systems where close to a billion transistors have to be interconnected in a area below 1 cm^2^, the low-*k* materials are needed as interlevel dielectrics (ILD) to minimize the effects caused by reduced line widths and minimal line-to-line spacings. The ILD material can decrease relative capacitance delay, cross-talk noise and power consumption [82]. It is especially important for fast signal propagation in high-speed electronic circuits [83]. These are the reasons that make the search for novel low-*k* materials of the main interest of numerous scientific groups.

The dielectric constant depends on molecular characteristics (polarizability α) and density (*N*), as can be concluded from the Clausius–Mossotti equation [84,85]. Polarizability allows for changing the *k*-value only in a limited range. Some polymers have good characteristics but exhibit insufficient mechanical properties and poor temperature stability [86]. Polymer-based materials are hardly compatible with current ultra-large-scale integration (ULSI) technology. A much more prominent decrease of the *k*-value can be achieved by changing the density of materials. Silica-based porous materials seem to be ideal for such an approach, which is reflected in numerous literature reports on that subject. Some the most interesting are described below.

The idea of inclusioning some air voids (the *k* value not much higher than 1) in materials was known before the development of stable mesoporous silica materials (just after MCM-41 development but still before SBA-15 era). For example, silica aerogel films exhibit low dielectric constant (<1.7) [87,88] which is comparable to silica xerogels where the dielectric constant was found to be even lower (approaching 1.4) [89]. The porous silica thin films, prepared by means of the surfactant-templated self-assembly method, was characterized by low *k*-value (below 2.5) as in the case of continuous cubic silica layers [90].

A similar approach was applied by Baskaran et al. [91]: the authors fabricated highly porous silica films with low dielectric constants in the range of 1.8–2.5. The materials were prepared by means of spin-on sol-gel process [92] using a polyoxyethylene ether surfactant to template nanometer-scale porosity with pore sizes of less than 5 nm. The limiting factor of those materials was related to interconnections which significantly hinders the practical application of the materials. 2D ordered mesoporous silica-based materials were much more promising as interlevel dielectrics due to the limited interconnections between pores. Just after the introduction of the SBA-15 silica, similar structures were applied as the materials with low k. Zhao et al. noticed their applicative potential virtually at the time of the first fabrication of SBA-15 [93]. The authors prepared and investigated various phases of porous silica thin films: 2D hexagonal, 3D hexagonal and 3D cubic. All samples were prepared with the use of non-ionic triblock copolymer as a template and dip-coating method in an acidic medium. The *k* value depends on the porosity of the film for pure porous silica materials. The measured *k* value varied from 1.45 to 2.10 for the investigated materials .

**Figure 4:**
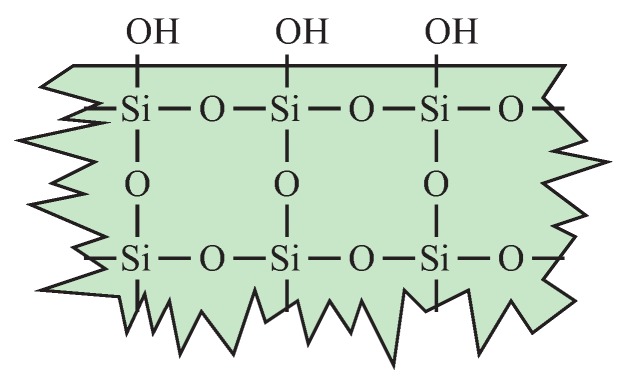
A schematic illustration of the silica structure with surface hydroxyl units. The structure is simplified and presented in a 2D plane.

The maximum porosity of the silica-based materials cannot be significantly increased while still keeping their good mechanical properties. One of the methods to obtain better dielectric properties seems to be the surface modification of materials [94]. The bulk of ideal silica is non-polar. However, numerous polar hydroxyl units are present on its surface (see Figure 4).

Polar units increase the value of a dielectric constant. Moreover, the larger the surface area is, the more polar hydroxyl units are in the material. Thus, an increase of porosity can decrease the *k*-value by including air voids but, at the same time, the number of hydroxyl units increases as well. The optimization of the silica structure towards low-*k* is therefore non-trivial. One solution to this issue can be hydrophobization of a surface by substitution of hydroxyl units by non-polar groups. Such an approach was applied in [95]. The authors prepared silica-based thin films by the spin coating method. The precursor solution was prepared using a triblock copolymer Pluronic P-123 (P123) as an organic template. Noteworthy techniques utilized by the authors are the application of the ozone ashing for surfactant removal and hydrophobization of the pores surface by means of silanization by hexamethyldisilazane. Substitution of the surface hydroxyl units by non-polar groups resulted in the decrease of dielectric constant to 1.8.

Similar results were shown in [96]. In this case porous silica, thin films were prepared also by means of spin coating but with the use of Brij30 structuring agent. After fabrication, porous thin layers underwent the vapor infiltration treatment using TEOS or trimethylethoxysilane (TMES). When TMES was applied, surface hydroxyl units were converted into non-polar trimethylsilane groups. This resulted in the decrease of dielectric constant to 1.8.

The TMES vapor treatment was applied also for unordered silica thin films. Maruo et al. presented an interesting procedure for the fabrication of wormhole-like porous silica by means of vapor phase method using TEOS as a silica precursor and F127 surfactant [97]. The post-synthesis treatment by vapor TMES allowed to achieve the dielectric constant even as low as 1.5.

**Figure 5:**
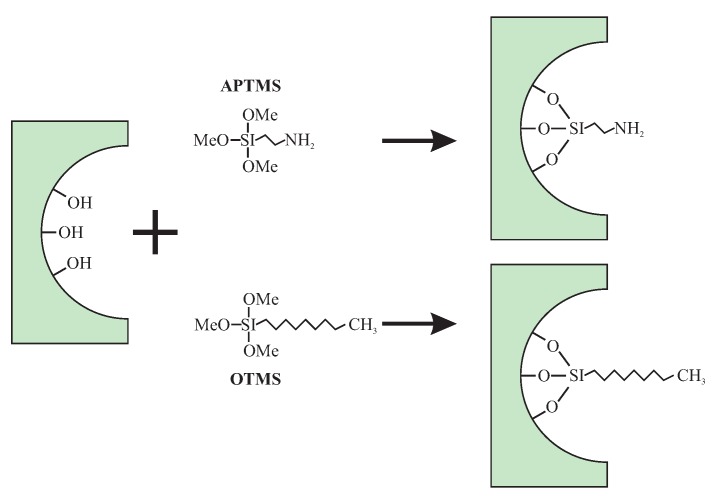
A schematic presentation of the silylation reaction presented in [98] and possible configuration of the SBA-15 after surface modification. Me, methyl groups.

Silica with a modified surface can ba a part of composite material together with a polymer. This kind of specimens is particularly important for the fabrication of flexible printed circuit boards and other microelectronic applications. This was presented by Min et al. [98]. The authors investigated composite materials composed of mesoporous silica (SBA-15) filled in polyamide. The materials were aimed to exhibit low dielectric constant and good mechanical properties. The SBA-15 were silylated with the use of two specimens: octyltrimethoxysilane (OTMS) or 3-aminopropyl trimethoxy silane (APTMS). This procedure resulted in the substitution of the surface hydroxyl units with two types of non-polar groups, as shown in Figure 5.

After the surface modification, specimens were coated with poly(amic acid) and investigated as such. The lowest observed *k*-value was 2.6 but optimal mechanical properties were obtained for the specimen composed of 97% of poly(amic acid) and 3% of modified SBA-15 when the *k*-value was approximately 3. The increasing of the SBA-15 content in a composite caused a decrease of the *k*-value. Nevertheless, the mechanical properties of the materials (tensile/elongation) were not satisfactory.

Much better results were presented in [99]. A somewhat similar material was presented based on benzoxazine functionalized SBA-15 silica (BZ/SBA-15). Similarly, the functionalized porous silica was coated by polybenzoxazine. Thus, the resulting composite material was composed of silica containing benzoxazine anchored inside pores and additionally covered with polymeric form of this dopant. Additionally, the amount of silica was very low: 2.5% and 7%. The composite containing 7% of BZ/SBA-15 showed a low dielectric constant of 1.73 with acceptable mechanical properties. Analogous research was also presented by other authors with similar results [100,101].

Presently, this technology is still being explored—another example can be found in [102]. The authors, similar to the previous cases, prepared a composite of SBA-15 with a polymer. The main difference was the functionalization of both the inner and the outer surfaces of porous silica by APTMS before coating. The author emphasized that aminopropyl groups are crucial for polymerization of the precursors into terpolyimide since the activated SBA-15 also took a place in the reaction. The value of the dielectric constant was moderately low: 2.42 but the proposed synthesis procedure was indeed interesting.

In the last years, functionalization procedures of porous silica-based materials were under rapid development. Precise loading of even complicated molecules inside pores became possible. Devaraju et al. showed the SBA-15-based material containing 3-glycidoxypropyltrimethoxysilane (GPTMS) located inside pores [103]. Such a modification induced a significant decrease of the dielectric constant from 3.34 for pure SBA-15 to 2.11 for the functionalized specimen. It was caused by the substitution of dipolar OH surface units on GPTMS, which has a high degree of symmetry in the triazine ring, where dipoles, associated with the carbon–nitrogen and carbon–oxygen bonds, are counterbalanced.

The methods quoted above were based on post-synthesis modification of the silica surface, mainly r to remove surface hydroxyl units and to create a hydrophobic material. A different approach to hydrophobization was shown by de Theije et al. [104]. The authors prepared porous silica-based thin films with controlled hydrophobicity by using the mixture of TEOS and MTMS as the silica precursors. To optimize the film properties and to check its influence on the dielectric parameters, three different surfactants were applied: CTAB, Brij76 and F127 (see abbreviations). It was possible to obtain well-structured thin films even in the case of the loading of 90% of MTMS in TEOS. Nevertheless, these mesoporous films containing even 50% MTMS in TEOS did not show the Si-OH signal and were hydrophobic for each of the used surfactants. Such an amount of MTMS was assumed to be optimal for low-*k* materials. Measurements of the dielectric constant showed values from 1.7 (for the thin film based on Brij76) to 2.0 (using CTAB). The intermediate value of 1.8 was obtained for the porous silica thin film fabricated with nonionic surfactant Pluronic F127. This film was the most stable, which was probably due to the increased wall thickness. It is worth emphasizing that loading MTMS caused partial crystallinity of the material, which is not common for mesoporous organosilica thin films.

Crystallinity can also influence the value of the dielectric constant [105]. Residual hydroxyl units can be found inside the silica structure, especially in amorphous phase, due to structural imperfections. The crystalline structure implies decreasing the $Q^{3}$, $Q^{2}$ and $Q^{1}$ numbers inside the silica structure, thus the number of OH units. This means that the dielectric constant can be decreased by increasing crystallinity. This idea was confirmed by Li et al. [106]. Pure silica zeolite thin films were synthesized by a two-step synthesis route. In the first step, the silica zeolite nanocrystals were prepared with the use of tetrabutylammonium hydroxide as the structure-directing agent and TEOS as the precursor of silica. Next, the thin films were obtained by spin coating of the zeolite nanoparticle suspension. The obtained materials showed high relative crystallinity (the particle size of approximately 50 nm), good mechanical strength and heat conductivity. The most important, however, was the value of dielectric constant: 1.5.

This idea was further developed and investigated by the authors of [107]. They compared thin films obtained by the method described above and by in situ crystallization method, which allows fabricating high crystallinity material and decreasing crystal defects. The pure silica zeolite single crystals showed simultaneously remarkably high elastic modulus (*E*) and low *k* values: *E* = 49.4 GPa and *k* = 1.78. Eslava et al. went further towards high elastic modulus and presented the study concerning pure silica zeolite layers with a bimodal pore size distribution [108]. Such materials are known to exhibit the highest ever reported elastic moduli [109]. The synthesis procedure was based on spin coating of nanoparticle suspensions, as in the above-quoted articles. The dielectric constant was kept below the value of 3. Further modification of the synthesis procedure [110] was realized by adding a solvent-evaporation process between the two thermal treatment steps. This allowed for obtaining much smaller particle sizes and maintaining the same nanocrystal yields as for the synthesis described before. The modification resulted in decreasing the *k*-value to 1.9.

**Figure 6:**
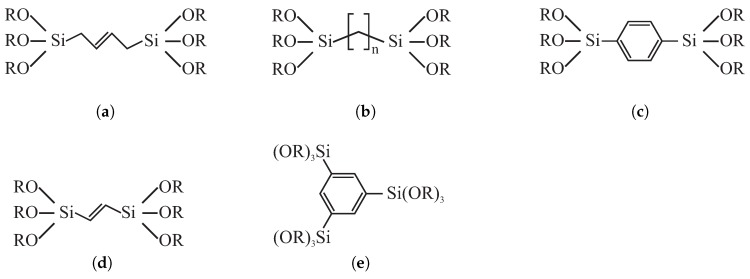
A schematic representation of the bridged silsesquioxane monomers applied in [111] ((**a**,**b**) for *n* = 2, 3, 6, 8, 10 (**c**)) and in [112] ((**b**) for *n* = 1, 2, (**c**–**e**)). R = C_2_H_5_.

The composition of silica walls can be modified by the inclusion of organic moieties inside silica structure. This way, a material can be obtained that is characterized by not only a low *k*-value but also additional functions. Lu et al. [111] presented evaporation-induced self-assembly procedure for the fabrication of the porous poly(bridged silsesquioxane) mesophases with integral organic functionalities that can be seen in Figure 6a–c.

The presented materials (prepared in the form of films and spherical nanoparticles) incorporated the organic constituents into the silica structure as molecularly dispersed bridging ligands. According to the authors, the introduction of integral organic groups into the frameworks of mesoporous materials resulted in the ability to tune their properties and function. The mechanical properties of the obtained materials were increased in comparison to the specimens without organic moieties while dielectric constant was kept in the range from 1.89 to 2.15.

Another example of the approach presented above can be found in the work of Hatton et al. [112]. The authors extended the range of silsesquioxane precursors (see Figure 6b–e) used for the fabrication of well-ordered porous thin films. Samples were prepared by means of spin coating of the solution of TMOS and silsesquioxane precursor in the presence of CTACl surfactant. In this case, the lowest *k*-value was 1.8.

The results presented above are not spectacular, however the direction seemed to be promising. Other scientists reached much lower value of the dielectric constant by applying similar approach. Yang et al. [113] prepared nanoporous organosilicates porous materials for application as an ultralow-*k* dielectric. The authors used hydrophobic poly-(methyl silsesquioxane) for the fabrication of a high-quality film by means of spin coating. In this case, the dielectric constant was as low as 1.5. Moreover, the mechanical properties were very promising allowing for heavy duty treatment.

Even perfectly designed and fabricated porous materials may behave correctly only in laboratory conditions. In real-life applications, however, the dielectric constant can be much higher than assumed. This can be caused by trapping moisture or gasses in pores or even some other contaminants. For this reason, the pore sealing seems to be essential for the porous materials to be applied as interlevel dielectrics [114,115]. A very interesting approach to this problem was presented by Jiang et al. [116]. The authors reported a plasma-assisted procedure allowing for atomic layer deposition (ALD) only at the immediate surface of the porous materials. This led to pore sealing at minimal thickness. Such a low penetration by sealing agent was achieved by the use of the “trigger” procedure: the non-reactive ADL was triggered by plasma, and then ALD could be spatially defined by the supply of plasma irradiation. The porous silica thin films were prepared by means of evaporation-induced self-assembly method with the use of Brij56 surfactant leading to continuous 3D arrangement of connected pores with diameters of 2 nm. Such films exhibited excellent mechanical strength and thermal stability along with a low *k* value. To seal the material, the authors proposed using silica, for which TEOS agent was used as a precursor. The atomic layer deposition was performed as a result of O_2_ and Ar plasma treatment. According to the authors, the associated radicals converted surface-adsorbed TEOS into reactive silanols and promoted further conversion to siloxane. This procedure led to the fabrication of almost ideal sealing. The silica covered the porous material with uniform thickness and no penetration into the porous matrix. As was shown, the plasma-activated atomic layer deposition of SiO_2_ as a coating resulted in a sufficiently dense and defect-free layer to seal the pores. It also protected the porous low-*k* silica from the exposure to gaseous chemicals. It is worth emphasizing that the authors presented the material with moderately low value of dielectric constant (*k* = 2.49) but in this case the key was the sealing procedure.

**Figure 7:**
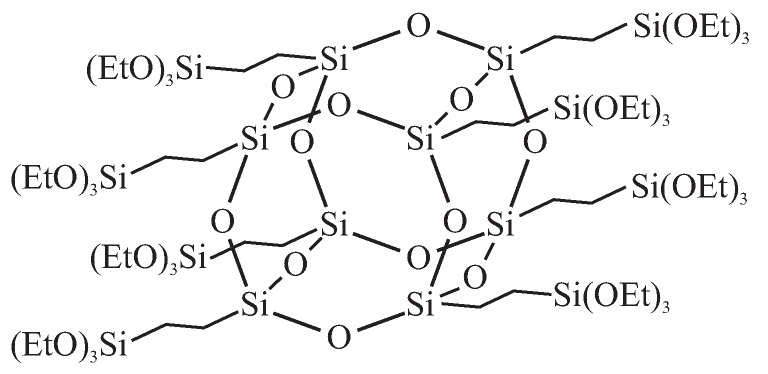
The cage-like structure of octa(triethoxysilylethyl)polyhedral oligomeric silsesquioxane. Et, ethyl groups.

A novel material, designed especially for application as a low-*k* dielectric, was presented by Seino et al. [117]: periodic mesoporous organosilica with polyhedral oligomeric silsesquioxane air pockets integrated into the pore walls. The material was synthesized with the use of octa(triethoxysilylethyl)polyhedral oligomeric silsesquioxane (OTES-POS) which has a cage-like structure containing silicon and oxygen with s covalently bonded reactive functionalities (OEt) suitable for polymerization or grafting (see Figure 7) [118].

By the application of the evaporation-induced self-assembly spin coating procedure (with CTACl used as a structuring agent), the authors obtained hexagonally 2D ordered mesoporous organosilica thin films. By the using the two-step surfactant removal procedure (washing by acidic etanole followed by calcination), the cage-like structure of polyhedral oligomeric silsesquioxane (POS) was kept intact and additional air voids were created in the structure of walls. This resulted in decreasing the *k*-value to 1.73 while maintaining the Young’s modulus of 3.30 GPa.

On the basis of the aforementioned research, one can conclude that the application of porous silica-based materials as a low-*k* dielectrics was thoroughly explored during the last two decades. Moreover, it is very hard to find a new article released starting from 2015 concerning this topic. It proves that the limit of the technology has been reached and further exploration of porous silica regarding the interlevel dielectrics makes no sense.

## 4. Redox-Active Silica-Based Organic-Inorganic Hybrids

As mentioned above, silica is an electronic insulator. Thus, its use in connection to electrochemistry has generated the search for strategies to confine silicates to electrode surfaces or to develop silica-based conductive composites. Such composites can be in the form of ceramic carbon composite electrodes or realized by incorporating noble metal nanoparticles into silica matrices to enhance their electrical conductivity [119,120,121,122,123,124]. Another approach relies on the concept of conducting polymer nanocomposites [125] and, especially, on the generation of interpenetrating silicate networks and conducting polymers [120,126]. The latter are prepared either via electropolymerization of pyrrole, aniline or thiophene monomers in the presence of silica precursors or nanoparticles [125,127,128] or by electropolymerizing the organic monomers into a preformed porous silica material [129,130] or even by co-electrodeposition [131]. A more elegant strategy is the use of a starting precursor bearing within the same molecule an alkoxysilyl part (likely to form the silica network) and a monomer part (likely to polymerize) to generate a hybrid material in which the conducting polymer was covalently bound to the silica [132]. This particular case has to be connected to a wider domain in which redox moieties are attached, in a covalent way, to silica frameworks. This is done to prepare the so-called “redox polymers”, which enable long-range charge propagation via electron hopping (or self-exchange) between adjacent redox centers with associated counterion diffusion to maintain electroneutrality [120,126]. They are usually prepared by post-grafting of a preformed porous silica material or in one step by co-condensation using organosilane reagents bearing the redox moieties (various examples are available, mainly for ferrocene, but also viologen, quinone or phenothiazine derivatives) [133,134,135,136,137,138]. However, the rates of charge transfer are usually slow requiring very high densities of redox moieties in the hybrid material to improve the efficiency of the electron hopping [133]. One way to circumvent this limitation is to add carbon/graphite particles to the organically modified silica to facilitate redox transformations of the immobilized centers via electron percolation through the composite material [137]. As shown below, based on more recent approaches, the regular and highly open structure of ordered mesoporous silica can contribute to significantly enhance such long-range charge transfer reaction in nanoporous silica-based materials.

Polymer-mesoporous silica nanocomposites can exhibit unusual properties offered by the advantageous combination of the attractive features of the ordered mesoporous host with the intrinsic characteristics of the accommodated macromolecules [139]. This is particularly true for conducting polymers for which physical encapsulation can contribute to the improvement of stability and possibly addressing individual molecular wires due to the separation of polymer chains and avoiding interchain effects [140]. Examples of conducting polymers confined in mesoporous silica for electrochemical purposes are available for polypyrrole [141,142] and polyaniline [143,144]. Covalent binding of the polymer wires to the silica walls is also possible, as firstly demonstrated for aniline-functionalized SBA-15 subjected to further polymerization in the presence of aniline gas [144]. Compared with bulk polymers, the structure and electrical properties of the macromolecular wires in the mesochannels are significantly modified after the formation of composites and their charge transport properties can be enhanced, offering potential applications as novel electronic or optoelectronic materials [145,146]. A straightforward way to prepare conducting polymer-mesoporous silica composites, exhibiting effective charge transport properties, is via electropolymerization of monomers (e.g., pyrrole, aniline or thiophene derivatives) inside the mesoporous structure. This is a method for which the growth of polymer chains can be, in principle, controlled by the electrochemical parameters and electrodeposition conditions. This is notably based on earlier works from Montilla et al. who reported the growth of polyaniline through porous sol-gel films on electrode surfaces [147,148]. The resulting materials exhibited attractive features in terms of enhanced capacitance [147] or improved electrocatalytic performance [149] relative to bulk polyaniline deposits. The approach can be extended to the electrochemical growth through ordered silica templates, leading to improved electronic conductivity and good electrocatalytic properties [150].

On the other hand, molecular redox-active moieties can be grafted to mesoporous silica materials, either into the mesopore walls [151,152] or onto the internal surface of mesochannels [153,154,155], and then to be exploited to facilitate long-range electronic transport phenomena in the insulating matrix, which could be expected to be more efficient in regular mesostructures than in non-ordered materials. A definite advantage of covalently anchoring the redox-active groups in comparison to simple doping (via weak bonds or electrostatic interactions) is a more durable immobilization (for instance, mesoporous silica films with embedded redox guest species such as [Ru(bpy)_3_]^2+^ are characterized by poor operational stability upon prolonged electrochemical cycling [156]). However, in the meantime, strongly attached redox probes do have intrinsically restricted motion possibilities which might result in impeded charge transport by electron hopping. This is especially the case of periodic mesoporous organosilica containing redox centers into the pore walls for which the electron transfer reactions are limited to the nanometer scale [151]. Efforts to circumvent this limitation are to either increase the redox groups content in the materials (but at the expense of the level of mesostructural order) [152], or electronically wire the redox-active centers via an intrapore conducting polymer [157]. The use of mesoporous silica with electroactive pendant groups, as obtained either by direct grafting [158] or via post-functionalization procedures [153], is offering much flexibility on terms of controlling the amount of immobilized species while maintaining a high degree of structural order. Most efficient charge transport is achieved for systems exhibiting high functionalization levels (i.e., high density of redox-active groups) promoting rather fast electron hopping between adjacent sites [153,158], using flexible arms to attach the electroactive groups to wide surface area silica walls (i.e., a large number of redox probes likely to “talk together”) [153,155], and ensuring good connection between the pores to facilitate charge propagation (and associated mass transport of counterions to maintain charge balance) in order to get the electron transfer reactions as deep as possible in the material [154].

**Figure 8:**
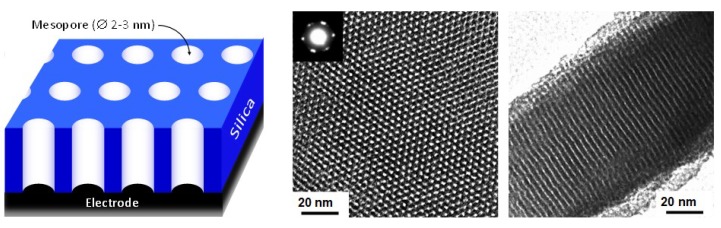
A schematic illustration of a vertically-oriented mesoporous silica film electrogenerated onto an electrode surface and typical corresponding TEM micrographs (top and cross-section views).

In respect to all above systems, the vertically aligned mesoporous silica films made of a hexagonal packing of mesopore channels all oriented normal to an underlying electrode support (Figure 8) [159] offer an ideal configuration to investigate the long-range charge transport phenomena in individual mesopore channels. These films can be generated by a versatile method called “Electrochemically Assisted Self-Assembly” (EASA) involving the vertical growth of silica walls around a cationic surfactant template under potentiostatic or galvanostatic control [40,160]. One already knows that such vertical orientation leads to fast mass transport of solution-phase redox probes through the film, resulting in highly sensitive electrochemical responses that can be exploited in the sensors field (e.g., [161,162,163,164]). We show next that these oriented films can also be used as template for conducting polymers and as support for the covalent binding of redox molecules, both giving rise to effective charge transfer with possible applications in sensing, electrocatalysis, energy or molecular electronics.

Pioneering works seeking to use vertically-oriented mesoporous films with hexagonal arrangement of cylindrical pores as hard template for the confined electrochemical growth of target redox-active nanowires have appeared since 2009, with examples involving the electrodeposition of Prussian Blue [165] or the electropolymerization of thiophene [166]. The presence of Prussian Blue in the film is confirmed by Energy-Dispersive Spectroscopy associated to Electron Microscopy and the resulting material is electroactive, although it probably does not fill totally the mesopore channels [165]. On the opposite, polythiophene nanowires of 6 nm in diameter are produced by electropolymerization [166], suggesting that the conducting polymer growth occurs also out of the film (as this value is larger than the mesopore diameter of 2–3 nm for such films). Template electropolymerization can be extended to the generation of other types of electronically-conductive nanowires, such as polypyrrole (PPy) [167], polyaniline (PANI) [168], poly(3,4-ethylenedioxythiophene) (PEDOT) [169] and other kinds of polythiophene [170], or even polyquinone [171]. A strategy to ensure strong and durable attachment of the nanowires onto the electrode surface is the formation of an underlying electrode previously modified with a thin film of the target conducting polymer onto which the mesoporous silica layer is formed and used as hard template for nanowires grown by in situ electropolymerization, remaining attached to the support even after template removal [169,172]. As pointed out for PANI, the growth of polymeric nanowires through the pores can be controlled by the experimental conditions (notably by tuning the electrodeposition parameters, in either potentiostatic or galvanostatic mode), and the resulting nanofilaments isolated from each other (thanks to the mesoporous silica template) exhibit considerably improved electrochemical reversibility (faster switching between doped and undoped states) in comparison to bulk PANI [168]. From charge–discharge measurements, it also appears that the high surface areas developed by the conducting polymer nanowires enable to reach extremely large capacitance values, by several orders of magnitude as compared to PANI or PPy films deposited in the absence of template [168,173]. To date, such nanocomposite films made of conducting polymers confined in oriented mesoporous silica films have been essentially applied for electrocatalysis and electroanalysis purposes [174,175,176], but they are also promising in the field of energy [168,173] as briefly mentioned above in the supercapacitors section.

Functionalization of vertically-oriented mesoporous silica films keeping accessible the selected functional groups offers great potentials in practical applications such as catalysis, adsorption, separation, sensing and nanotechnology. First attempts to afford functionalized vertically-oriented silica thin films are based on co-condensation of an alkoxysilane and an organosilane. This is however restricted to simple organic functional groups such as alkyl [177,178], thiol [179], and/or amine [180] moieties, which are usually incorporated in limited amounts into the final material. Introduction of larger amounts and more complex organic functions usually leads to the degradation of the mesostructure and lost of the vertical orientation. The combination of the electrochemically-assisted deposition of clickable ordered and oriented azide-functionalized mesoporous silica with alkyne-azide click chemistry has enabled more recently getting such nanostructured and vertically-aligned hybrid films bearing significant amounts of more sophisticated organic moieties [181]. The feasibility of the click reaction and versatility of the approach in the confined space have been demonstrated by employing several molecules bearing a terminal alkyne function such as ethynylferrocene [181,182], propargyl alcohol [183], ethynylpyridine [181] and propargyl tetrazine [184]. In the following, we focus exclusively on the films bearing redox-active moieties likely to induce long-range charge transfer through the insulating porous silica layer.

The first examples of silica-based hybrid redox polymers are based on ferrocene-functionalized sol-gel materials [134,185,186,187]. In general, such materials exhibit moderately fast electron transfer kinetics. However, due to the confined state of redox centers linked to the polymeric backbone, charge transfer processes occur mainly via electron hopping mechanism between adjacent sites and many experimental parameters are likely to affect the electrochemical response of such hybrid redox-polymer modified electrodes. In particular, the electron transfer rate is strongly influenced by the rigid structure forced by the xerogel leading to significant decrease in the electrochemical response due to restrained movement of the redox sites and by the composition (redox sites concentration, etc.). Concerning the functionalized silica thin films bearing electroactive species, careful analysis of the electrochemical response, in particular of the ferrocene-functionalized silica films, indicates a strong dependence not only on the density of redox moieties covalently attached to the mesoporous walls but also on the supporting electrolyte [182]. The redox response results from a compromise between fast electron hopping (favored for high ferrocene amounts) and fast mass transport of the anions for charge compensation (favored at low ferrocene contents). The long-range charge transfer by electron hopping has been unambiguously evidenced by using a size-excluded nona-ferrocenyl dendrimer in solution with a ferrocene-functionalized film with a pore diameter of 2 nm; this system is likely to carry electrons between the electrode and size-excluded molecules over 100 nm distance [188]. As a consequence, the electrochemical communication between the dendrimers is determined by the electron transport rates through the film acting as a redox polymer.

**Figure 9:**
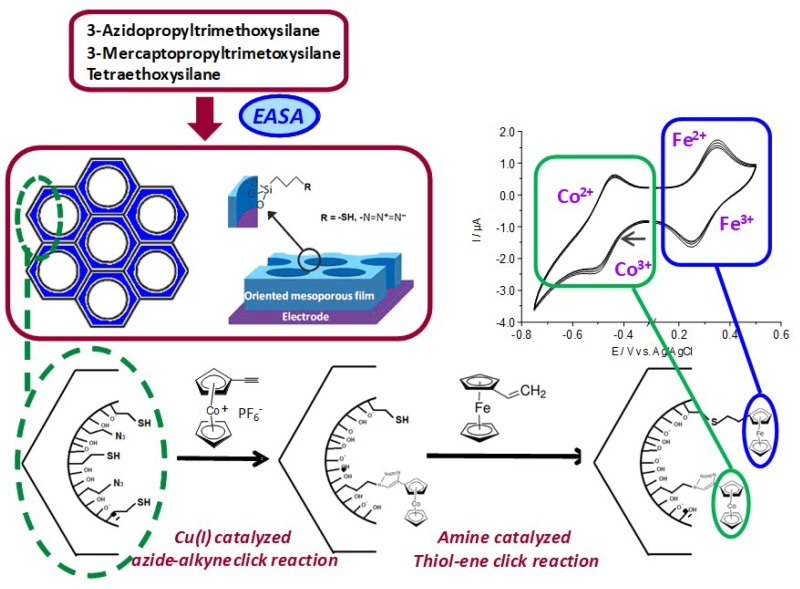
An illustration of the elaboration of oriented azide-thiol-functionalized mesoporous silica film by EASA and subsequent derivatization of azide groups by reaction with an alkyne derivative and the thiol ones with an alkene one.

The methodology primarily developed to afford monofunctionalized silica thin films can be further extended to the preparation of bifunctionalized films based on a versatile, generalizable and selective route to perpendicularly oriented and bifunctionalized mesoporous silica thin films involving a dual click chemistry approach combined to the EASA method [183]. To this end, EASA is first used to generate highly ordered and vertically oriented mesoporous thin films bearing large amounts of both azidopropyl and mercaptopropyl groups. They are then exploited as clickable moieties in a double sequential stepwise post-functionalization process to get the final targeted organic functions on the material. The azide and thiol groups are likely to react with a variety of organic molecules possessing, respectively, an alkyne or alkene terminal function, opening the door to the dimensional confinement of many functional groups in such hybrid films with perpendicular mesochannels (Figure 9). The feasibility of this simple and generic strategy is illustrated here for a couple of model derivatives (ethynylcobaltocenium/vinylferrocene), giving rise to well-defined cyclic voltammetry responses, but such stepwise post-functionalization can be extensively used for the incorporation of a variety of other organic functions in mesoporous silica films. To our knowledge, this is the first example of a selective bifunctionalization of vertically aligned silica thin films by a versatile sequential post-functionalization strategy which can be applied as dual clickable platform to introduce a wide range of organic functions in such mesoporous films with finely tunable properties thanks to the numerous possibilities offered by such coupling reactions.

These organic–inorganic hybrid mesoporous silica films are promising for applications in molecular electronics, especially in devices requiring long-range charge propagation at the nanoscale through isolating coatings. They are also of interest for the development of electrochemical sensors and in the field of energy [189]. Note that mesoporous silica-based materials have long-lasting interest in applications for sensing changes in relative humidity, changes in pH, metal cations, toxic industrial compounds, volatile organic compounds, small molecules and ions, nitroenergetic compounds, and biologically relevant molecules, as extensively reviewed by Melde et al. [190]. Their modification by introducing reactive organic moieties in the mesoporous silica-based materials, resulting in organic-inorganic hybrids, has generated even more possibilities and performance improvements in the design of electrochemical sensors, notably for preconcentration electroanalysis of organic and inorganic pollutants, electrocatalytic detectors and amperometric biosensors, as reported in some comprehensive reviews [191,192].

## 5. Perspectives

All the applications described in the previous sections can be treated as closed chapters: supercapacitors, low-*k* materials and hybrids based on porous silica are thoroughly explored from the scientific point of view. We do not expect any breakthroughs in the near future for the first two fields, but we are confident that the numerous possibilities offered by multiple functionalization of mesoporous silica films with organic groups would lead to significant advances in molecular electronics by selecting the most appropriate charge transfer mediators. On the other hand, there still exist unexplored applications for which mesoporous silica seems to be ideal. In this section, we present two of them as the marker of the direction for future research.

The first concept material worthy of presentation is molecule-based realization of Hopield-like artificial neural networks: the Molecular Neural Network (MNN).

**Figure 10:**
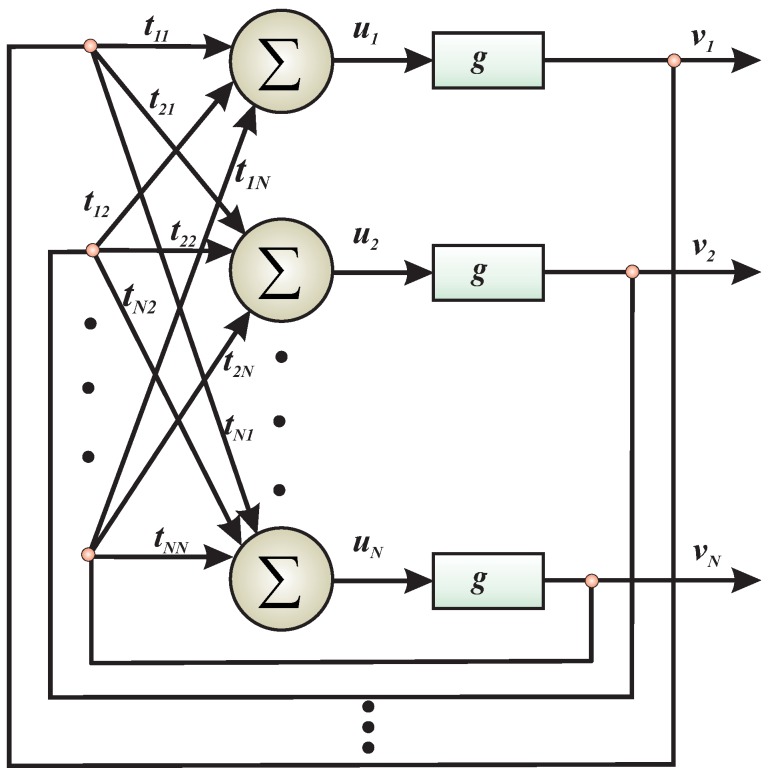
The structure of the Hopfield-like continuous neural network. In the picture, $t_{ik}$ is an interconnection strength between the neuron *i* and *k*, $u_{i}$ is internal potential of neuron *i*, and *g* is a continuous activation function (usually sigmoidal) with given external potentials $v_{i}=g\left(u_{i}\right)$.

In its original form, the Hopfield neural network was introduced in 1982 by J. J. Hopfield [193]. Hopfield’s original concept of neural computation was a ground-breaking idea in the neural networks domain, allowing for the construction of auto-associative memories [194] or systems for multi-criterion optimization [195,196]. Contrary to existing artificial neural networks, Hopfield’s structure was no imitation of biological neural systems but a computer simulation of a spin-glass [197]. The most important feature of spin-glasses, as far as Hopfield architecture is concerned, is their complex energy landscape with numerous local minima [198]. Such systems are subjects to Minimum Energy Law (as all physical systems)—only the minimum energy configuration is stable. Such systems show a slow drift towards their minimum of the energy function (Hamiltonian). In the spin-glass, the energy landscape is fully determined by the value of exchange interactions between contributed atomic spins. The evolution of the system depends on continuous adjustment of the atom spins orientation in response to magnetic fields originating either from other atoms or external ones. As any other physical system, spin-glass also continuously approaches its minimum energy configuration during its evolution. Such a configuration is stable and does not change in time. Thus, spin-glass has a property of a total energy minimization (Hamilton function)—the only possible stable state of thpie atomic spins corresponds to the local energy minimum. Interestingly, this property can be used to build neural computational systems. Hopfield’s idea was simple: the description of a problem can be given by the couplings (or so-called interconnection strengths) in the sense that the energy minimum corresponds to the solution (where the interconnections create the energy landscape), i.e. the solution in the form of the minimum-energy configuration of a spin-glass is found as a result of its relaxation. His computer realization of the spin-glass involved substitution of atoms by neurons, exchanging interactions by interconnections strengths and creating neural networks, as shown in Figure 10.

To solve the optimization problem by a Hopfield Neural network, the problem *A* must be written in a proper form. Conditions that must be fulfilled can be written as follows:

1. The problem, must be formulated in such a way that each syntactically correct solution corresponds to the local energy minima.
2. A lower energy minimum corresponds to s better solution.
3. The best solution corresponds to the global minimum.

It should be expressed in the square form allowing for calculation of the interconnection strengths by comparison of the problem formulation to the Hamilton function, similar to for the spin-glass. The transformation of optimization problem *A* into s form that would be accepted by Hopfield neural network depends on selecting of interconnection strengths $t_{ij}$ in such a way that the solution of the problem represents energy minima fulfilling Conditions 1–3. Then, the problem can be solved by the neural network during its relaxation: the network “points out” the position of the energy minimum by the configuration of the external states $v_{i}$. A detailed description of the minimization procedure by using Hopfield neural network can be found elsewhere [199].

The idea was revolutionary. However, thus far some practical implementations of this idea have not met the expectations. In many fields (e.g., associative memories), Hopfield networks have been applied providing the best solution. Nevertheless, due to the constraints of these systems, they could only mimic the original Hopfield’s concept. In the real working conditions, the computer simulations of the Hopfield neural networks stacked in local minima, shows errors connected with discretization and worked in a such a slow manner that using of the neural algorithm in a real-time working devices was impossible. All these problems originate from one important feature: Hopfield-like networks only imitates spin-glass.

The solution could be a “step back” to original spin-glasses as they can operate in a parallel way. Unfortunately, its implementation is challenging since both the determination of couplings between neurons and checking the spin states of neurons are hardly possible. Nevertheless, all these problems can be solved by the enlargement of the spin-glass to the dimension giving possibility of reading the configuration of atoms/nodes/neurons (next called processing units) and setting interconnections strengths between processing units (energy landscape shaping).

In this way, we can precisely define the necessity: the layout of small bistable units connected in such a way that they can interact with each other (parallel continuous operating). At the same time, processing units should be large enough to allow reading their configuration and controlling interconnections. Processing units should be distributed in a 2D regular way. The regularity of distribution facilitates localization, connections and states reading. Such a layout could operate much faster and more precisely as a computer simulation of a Hopfield network. Such a proposed device can work as spin-glass and not only imitate it.

Proposed device can be based on vertically-aligned mesoporous silica thin film. There, porous layer is a template allowing for the regular distribution of the magnetic molecules in the roles of processing units. Moreover, silica walls shield magnetic interactions between neighboring processing units. We propose using the electro-assisted self-assembly method for fabrication of the silica template [40] to obtain thin silica layers containing highly 2D ordered vertically aligned channels, as described in previous section (see Figure 8). Each pore filled by some magnetic molecule behaves as a bistable (or sigmoidal) processing units. Taking under consideration the geometry of such thin film, it is possible to obtain huge neurons density: 13 × ∼10^10^ units in mm^2^. Now, one of the most important question is: How can processing units inside silica pores be achieved? This can be done by a very precise functionalization of the pores bottom by single-molecule magnets with assumed magnetic and structural properties. We aim for molecules with relatively high ground spin and dimensions little lower than the diameter of the pores. High magnetic moment allows for reading of the magnetic states of molecules (MFM or XMCD). Dimensions close to the diameter of the pores assure that each pore will contain only one magnetic molecule anchored at the bottom. The ideal candidate seems to be Mn_12_-stearate single molecule magnet [200]—a soluble derivative of Mn_12_-ac_16_ [201]. The manganese core shows a high intrinsic spin (S = 10) and a slow relaxation of magnetization [202].

**Figure 11:**
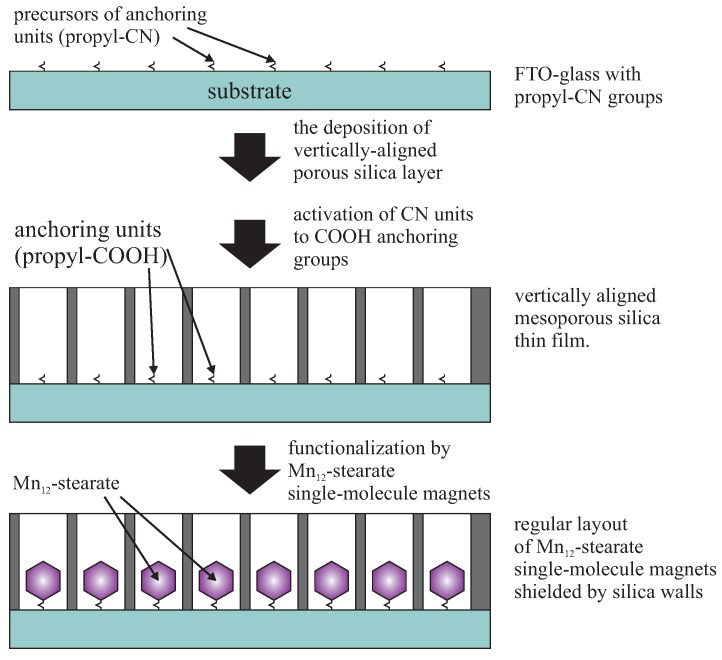
A schematic representation of the synthesis procedure for fabrication of vertically aligned thin mesoporous silica layers containing singular Mn_12_-stearate molecules at the bottom of channels.

Other important questions arise: How can such a precise functionalization be performed? How can a single Mn_12_-stearate molecule in each pore be obtained? The method which makes this possible is a multi-step functionalization (MSF) procedure that has been already tested with good results [203]: we have shown that it is possible to anchor the metal ions exclusively at the pores bottom. The procedure can be applied also for functionalization by Mn_12_-stearate molecule. Taking under consideration dimensions of these SMMs (a shape that can be inscribed into a flattened ellipsoid with dimensions of 1.5, 2.5 and 3 nm depending on the observation angle), the application of MSF assures the anchoring of single SMM inside each pore, as shown in Figure 11.

**Figure 12:**
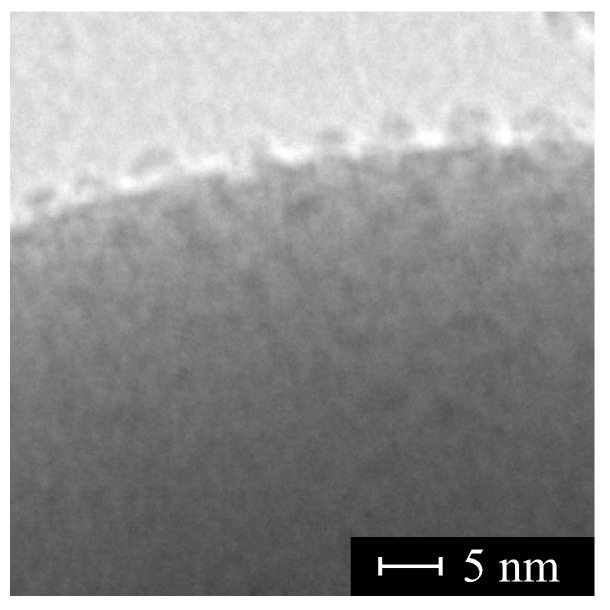
The transmission electron micrographs of the individual Mn_12_-stearate molecules attached to the spherical silica surface.

It may be doubtful whether or not it is possible to separate and attach the Mn_12_-stearate SMMs but this possibility was also tested and directly observed under TEM (Mn_12_-st were separated onto spherical silica surface (see Figure 12) [204].

**Figure 13:**
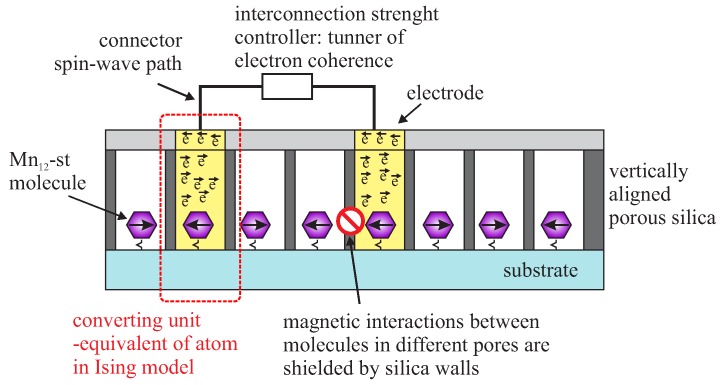
A schematic illustration of the molecular neural network with two interacting molecular neurons (processing units).

Looking at the preliminary results and the initial research presented above, we are convinced that preparation of the regular layout of the independent magnetic units is feasible. Surely, such a device will be operating only at a low temperatures below the blocking point (3 K). Now, we face one important problem: how to connect the processing units and how to control the interconnection strengths. Here, we can only propose a solution. In our opinion, the control of processing units interactions between each other can be realized through Fermi electrons as in the case of spin-glass [198]. Exchange strengths can be tuned by adjusting the coherence of electrons. Obviously, the coherent way of electron is much shorter than the distance between processing units. However. this problem can potentially be solved by conversion into a spin-wave [205] and using a Datta–Das [206] transistor for coherence adjusting, as shown in Figure 13.

Here, we must remark that the connection of processing units can be an extremely difficult task—there are numerous problems to overcome. Nevertheless, in our opinion, scientific research should give the possibility to go beyond assumed frameworks or, actually, even broaden all the frameworks and limitations. The concept of molecular neural networks seems to be worth of extensive scientific efforts.

**Figure 14:**
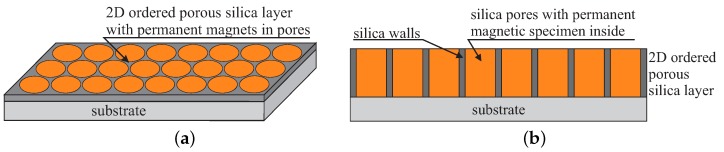
The structure of the silica-based memory storage device: perspective projection (**a**); and cross section (**b**).

A somewhat anologous and simplified concept can be used for the construction of super-dense magnetic memory storage device. Nevertheless, we must mention that simplified does not mean easy to fabricate. The concept device can be defined as a regular layout of separate, independent permanent magnetic units with the diameter of approximately 2 nm. Additionally, the units should be separated from each other by silica walls with the thickness of 1 nm. The magnetic units are placed inside vertically aligned (perpendicularly to the substrate) silica pores arranged 2D hexagonally, as shown in Figure 14.

**Figure 15:**
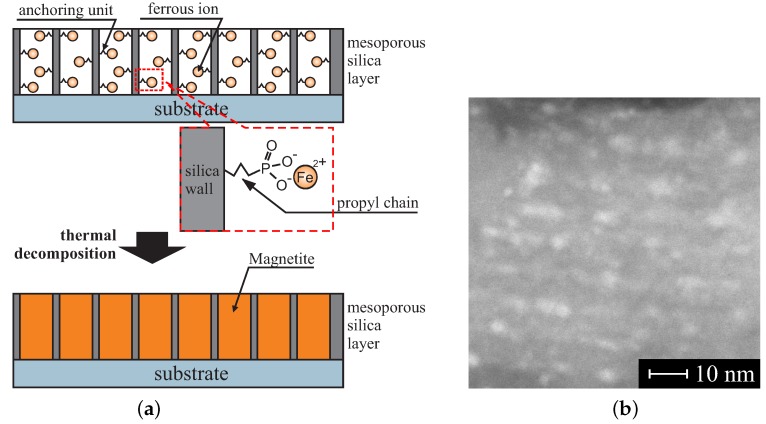
The general illustration the procedure of magnetite synthesis by the use of silica nanoreactors (**a**); and resulting nanoparticles of magnetite obtained inside SBA-15 (TEM image) (**b**).

The thickness of such a layer is in the range of 70–100 nm. We assume that magnetic states of the units can be kept at room temperature. Similarly, this task also seems to be extremely difficult. How can permanent magnets operating in room temperature inside the pores of diameter of 2 nm be fabricated? According to our preliminary research, it is possible. The silica pores play the role of dishes that can be filled by a permanent magnetic specimen: magnetite (Fe_3_O_4_). Mesochannels can be considered as nano-reactors enabling super-precise control of the concentration of reagents. This can be done by precise functionalization of the pores interiors by ferrous ions [207]. Assuming the correct concentration of the internal propyl iron phosphonate units, it is possible to create magnetite nanocrystals inside nanoreactors as a result of a thermal decomposition by precisely controlling selected heating parameters (temperature, heating rate and atmosphere), as shown in Figure 15a.

We tested the procedure with the use of SBA-15 silica as a matrix—powdered form of silica allowed for convenient performing of research. We obtained fine nano-crystals inside silica pores with diameter of below 4.7 nm (see Figure 15b). The crystals were identified as a magnetite. Moreover, they showed magnetic hysteresis at the room temperature despite their low diameters (below super-paramagnetic limit). We admit we are not entirely sure of the reasons for this phenomenon. Currently, we can only theorize that it is a result of the spatial confinements (article under preparation). Nevertheless, all the results shoved us that the fabrication of the porous silica-based super dense memory storage system is possible.

Both concepts presented above are challenging for realization. However, looking at the preliminary results we, are convinced about their feasibility. Moreover, their applicative potential seems to justify all scientific efforts toward fabrication of the presented devices.

## 6. Conclusions

Over the years, electronics have become a larger and larger part of our lives to the point where we stop noticing them. The behind-the-scenes work of researchers to keep improving them described here shines a light on the complex mechanisms involved in this process. Understanding these phenomena allows for searching for novel materials applicable in electronics.

On the brink of the end of the silicon era, mesoporous silica is potentially the next standard as the base material used in electronics. Tailorable properties through different routes of the bottom-up syntheses, flexible functionalization and the new emerging fields of science in which the material can be applied make mesoporous silica-based materials an exciting alternative.

Here, we describe this material in such a context. We present d the development and most crucial findings of mesoporous silica as far as electronic devices are concerned. Porous silica are commonly used in several domains, including an industrial use. Obviously, there also exist less explored domains where these porous materials have just been introduced.

Concerning the former case, supercapacitors, low-*k* materials or sensors are good examples. Here, porous silica is an evident material for the fabrication of electrodes or interlevel dielectrics. This field is well described by numerous publications including some reviews.

The latter case concerns emerging technologies. Here, the literature coverage is lacking. We present a few examples of such possible applications: the molecular neural network and silica-base super-dense magnetic memory storage. It can be treated as a preliminary communication on concept devices or as inspiration for further scientific works.

The demand will drive the supply and new exciting discoveries in the field of electronics are sure to come. Indeed, scientists even search for more and more sophisticated devices to conduct research.

## Abbreviations

The following abbreviations are used in this manuscript:

TEOS	tetraethoxy silane
TMOS	tetramethoxy silane
TMES	trimethylethoxysilane
MTMS	methyltrimethoxy silane
OTMS	octyltrimethoxy silane
APTMS	3-aminopropyl trimethoxy silane
Me	methyl units
Et	ethyl units
CTAB	$C_{16}H_{33}-N{\left(CH_{3}\right)}_{3}^{+}Br^{-}$
CTACl	$C_{16}H_{33}-N{\left(CH_{3}\right)}_{3}^{+}Cl^{-}$
Brij76	$C_{18}H_{37}{\left(OCH_{2}CH_{2}\right)}_{10}OH$
Brij56	$C_{16}H_{33}{\left(OCH_{2}CH_{2}\right)}_{10}OH$
Brij30	$C_{12}H_{25}{\left(OCH_{2}CH_{2}\right)}_{4}OH$
Pluronic F127	poly(ethylene oxide)-poly(propylene oxide)-poly(ethylene oxide) triblock copolymer:
	PEO_106_-PPO_70_-PEO_106_
Pluronic P123	poly(ethylene oxide)-poly(propylene oxide)-poly(ethylene oxide) triblock copolymer:
	PEO_20_-PPO_70_-PEO_20_
ALD	atomic layer deposition
OTES-POS	octa(triethoxysilylethyl)polyhedral oligomeric silsesquioxane
MFM	magnetic force microscopy
XMCD	X-ray magnetic circular dichroism
SMM	single-molecule magnet
Mn_12_-ac_16_	$\left[Mn_{12}O_{12}{\left(CH_{3}{\left(CH_{2}\right)}_{16}CO_{2}\right)}_{11}{\left(CH_{3}CO_{2}\right)}_{5}{\left(H_{2}O\right)}_{4}\right]\cdot 2CH_{3}COOH$
Mn_12_-st	derivative of Mn_12_ containing strearic acid ligands
MSF	multi-step functionalization procedure
